# E-learning or educational leaflet: does it make a difference in oral health promotion? A clustered randomized trial

**DOI:** 10.1186/s12903-018-0540-4

**Published:** 2018-05-10

**Authors:** Susan Al Bardaweel, Mayssoon Dashash

**Affiliations:** Department of Paediatric Dentistry, Faculty of Dentistry, Damascus University, Damascus city, Syria

**Keywords:** Health education, Knowledge, Oral health promotion, E-learning, Leaflets, Schoolchildren, Syria

## Abstract

**Background:**

The early recognition of technology together with great ability to use computers and smart systems have promoted researchers to investigate the possibilities of utilizing technology for improving health care in children. The aim of this study was to compare between the traditional educational leaflets and E-applications in improving oral health knowledge, oral hygiene and gingival health in schoolchildren of Damascus city, Syria.

**Methods:**

A clustered randomized controlled trial at two public primary schools was performed. About 220 schoolchildren aged 10–11 years were included in this study and grouped into two clusters. Children in Leaflet cluster received oral health education through leaflets, while children in E-learning cluster received oral health education through an E-learning program. A questionnaire was designed to register information related to oral health knowledge and to record Plaque and Gingival indices. Questionnaire administration and clinical assessment were undertaken at baseline, 6 and at 12 weeks of oral health education. Data was analysed using one way repeated measures ANOVA, post hoc Bonferroni test and independent samples t-test.

**Results:**

Leaflet cluster (107 participants) had statistically significant better oral health knowledge than E-learning cluster (104 participants) at 6 weeks (*P* < 0.05) and at 12 weeks (*P* < 0.05) (Leaflet cluster:100 participants, E-learning cluster:100 participants). The mean knowledge gain compared to baseline was higher in Leaflet cluster than in E-learning cluster. A significant reduction in the PI means at 6 weeks and 12 weeks was observed in both clusters (*P* < 0.05) when compared to baseline. Children in Leaflet cluster had significantly less plaque than those in E-learning cluster at 6 weeks (*P* < 0.05) and at 12 weeks (*P* < 0.05). Similarly, a significant reduction in the GI means at 6 weeks and 12 weeks was observed in both clusters when compared to baseline (*P* < 0.05). Children in Leaflet cluster had statistically significant better gingival health than E-learning cluster at 6 weeks (*P* < 0.05) and 12 weeks (*P* < 0.05).

**Conclusions:**

Traditional educational leaflets are an effective tool in the improvement of both oral health knowledge as well as clinical indices of oral hygiene and care among Syrian children. Leaflets can be used in school-based oral health education for a positive outcome.

**Trial registration:**

Australian New Zealand Clinical Trials Registry (ACTRN12618000395235), Date registered: 16/03/2018, retrospectively registered.

**Electronic supplementary material:**

The online version of this article (10.1186/s12903-018-0540-4) contains supplementary material, which is available to authorized users.

## Background

Due to the tremendous increase in the use of new technologies, it is thought that younger generations think and process information in a different manner than their predecessors [1]. Technology has changed the way we see the world [2]. There are many definitions of E-learning, one of these definitions is the use of “Internet technologies to deliver a broad array of solutions that enhance knowledge and performance” [3]. However, E-learning is a broad term that includes any use of computers to support learning process, whether online or offline [4].

School age is influential in people’s lives. It is a time when lifelong sustainable oral health related behaviors, beliefs and attitudes are being instilled. During this stage, children are more receptive; in addition, earlier establishment of habits produces a longer lasting impact. Therefore, schools can be considered an ideal environment for promoting oral health [5].

Dental caries has been considered to be a major public health problem for Syrian children. Despite a significant increase in the number of dentists in Damascus city, epidemiological data did not indicate any decrease in the dmft (decayed, missing and filled primary teeth) or DMFT (decayed, missing and filled permanent teeth) values for any age group. Additionally, no decrease in the percentage of untreated dental caries is detected [6]. Moreover, many challenges can be faced in providing access and delivering oral health care to children in Syria. Therefore, it is thought wise to increase preventative care in the form of school-based health education programs aiming at children.

Researchers have measured the effectiveness of E-learning in different areas. However, there are no previous studies that compare the effects of two different educational methods (E-learning versus leaflets) on oral health promotion geared for school children. Our intention was to enhance the application of evidence so, to minimize contamination, the unit of randomization was the school. The present study aimed to determine if E-learning instructions improve the acquisition of oral health knowledge and skills when compared to traditional educational leaflets in children aged 10–11 years living in Damascus city. Also, to consequently determine which educational method can better direct the child towards practicing appropriate oral health care.

## Methods

### Study design

A clustered randomized controlled trial at two public primary schools of Damascus city, Syria, was conducted. Using a list of the public schools in the city of Damascus, two schools were randomly selected by simple random sampling method using the table of random numbers; geographic location was taken into consideration in order to minimize any unintentional spillover effect of the assigned intervention. For allocation of the schools, simple randomization through flipping a coin was used by an investigator with no clinical involvement in the trial. The two schools were randomly allocated into two clusters: Leaflet cluster included 110 children, who received oral health education through leaflets, and E-learning cluster included 110 children who received oral health education through an E-learning program. The whole study was carried out for a period of 3 months (February 2016–April 2016).

### Sample

An estimate of 100 subjects per cluster was calculated to detect a difference between the two clusters with a two-tailed, α of 0.05 and a (1-β) of 0.80. Common occurrences such as loss to follow-up, missing data and withdrawals from the experiment were anticipated and additional subjects were recruited into each cluster. Initially 247 children from two public primary schools were asked to participate in the study. Informed consent was obtained from the parents of 220 with 110 children in each cluster who were initially included in the study, and then sample diminished through the recall visits due to children having moved to other schools or being absent the day of the examination. Thus the final sample included 200 with 100 children in each cluster (91 boys and 109 girls) aged 10–11 year old.

### Inclusion criteria

All healthy children who accepted to take part in this study, who did not receive any previous dental educational program, had internet access connection and ability to browse and use the internet, were included in this study.

### Exclusion criteria

The following groups were excluded: children outside the age range of the study; children currently under the regular care of an oral health care provider; children who have access to oral health education through a different and separate source than our intervention; children with acute dental issues (e.g.: dental abscess); mentally or physically compromised children and finally children whose parents did not provide consent for participation in our study.

### Ethical considerations

Ethical Approval was obtained from the ethics committee of the Faculty of Dentistry in Damascus University, Syria. In addition, a formal permission was obtained from the Ministry of Education in order to get access to schools and perform the required examinations on children. A written informed consent was obtained from all parents of the study participants.

### Educational tools

**Fig. 1:**
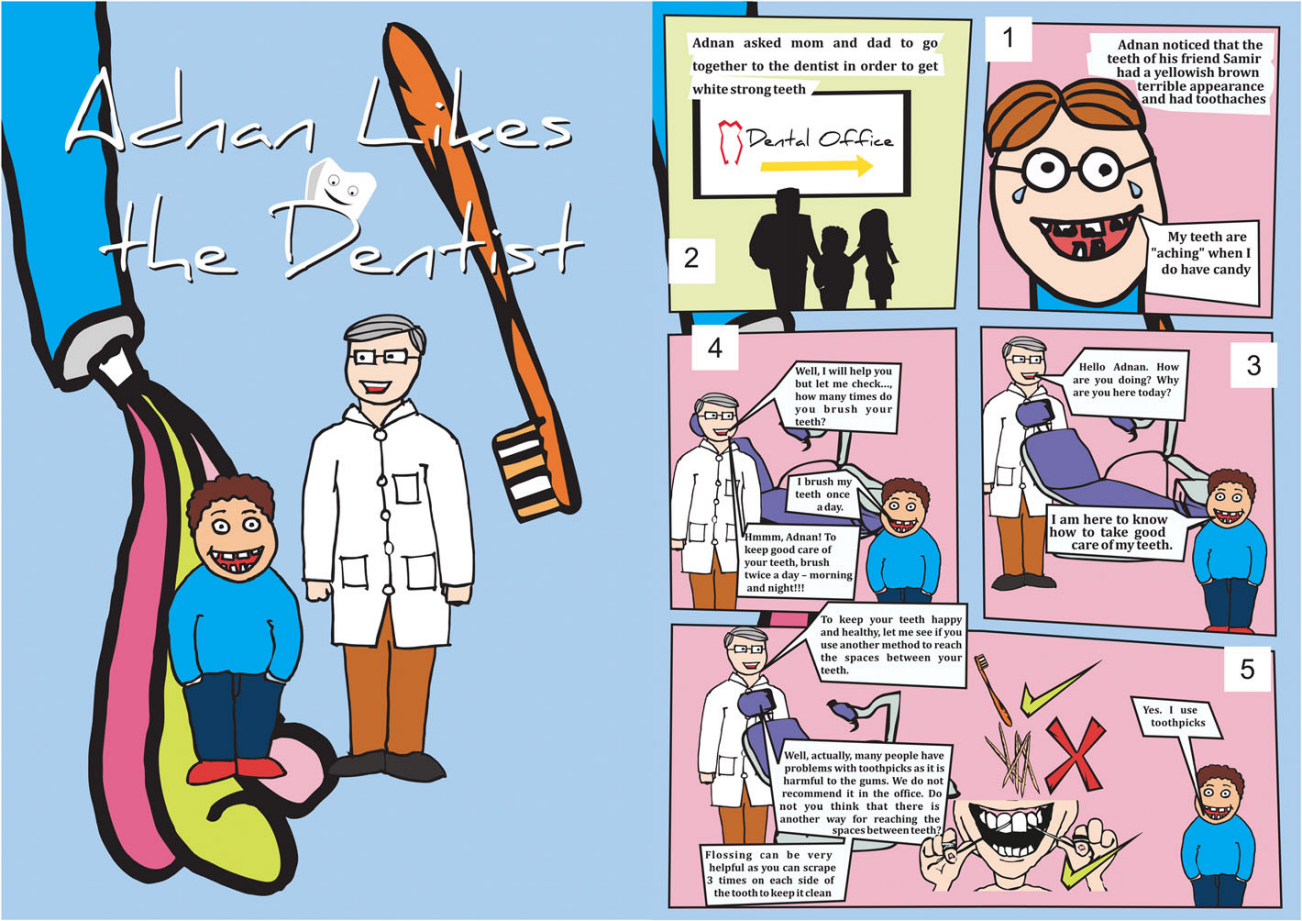
Shows the first part of the educational leaflet

**Fig. 2:**
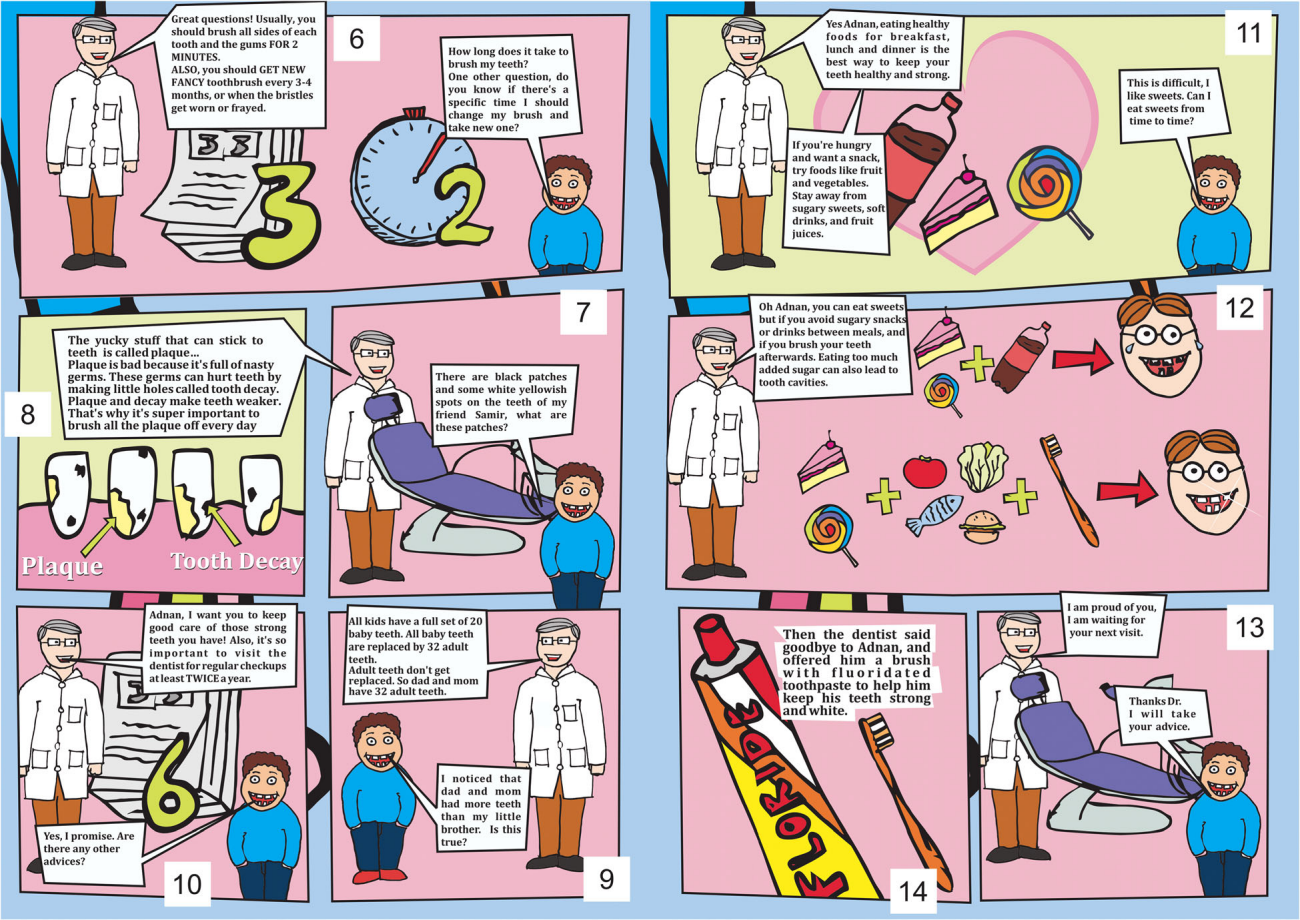
Shows the second part of the educational leaflet

*Leaflets:* A colorful and attractive leaflet in the form of a short story named “Adnan likes the dentist” was designed by a graphic designer (Figs. 1 and 2). The leaflets were designed with particular emphasis on creating interest amongst the children. These educational papers included information related to proper brushing technique and frequency; introduced the regular use of dental floss; emphasized regular dental visits as well as provided basic demonstration of dental plaque and the implications of not removing it. The leaflets also contained nutritional guidelines in regards to minimizing caries risk, and finally the role of fluoride in caries control.

#### E-learning program

An E-learning program was designed by an expert in artificial intelligence. The program was full of colorful images, videos, interactive quizzes and age-related developmental tasks in the quest to deliver the information in an interactive, entertaining and simple manner. The E-learning program included the same information of the leaflet; only the way in which the content is conveyed to the children was different.

### Questionnaire design

Several issues were considered essential in designing the questionnaire for the children aged 10–11 years. The developmental age of children was the key in order to provide them with age–related developmental tasks that can offer educational opportunities and tools for health promotion and encourage children to use and maintain their oral health. Therefore, a panel of experts from the Faculties of Education and Dentistry were consulted in order to design the questionnaire.

Information related to oral health and nutrition was included. A pilot study which included 25 children was undertaken to identify any ambiguous or unclear terms, and to assess the time required for filling the questionnaire.

The final draft of the designed questionnaire included demographic data such as name, age and school name. It also included charts for recording Plaque and Gingival indices and simple Arabic questions to assess knowledge, practices of oral health and diet (Additional file 1).

### Study procedures

The two selected schools were randomly allocated into two clusters: Children in Leaflet cluster received oral health education through leaflets, and children in E-learning cluster received oral health education through an E-learning program. Only one trained investigator (S.B) clinically examined all children in their classroom using mirror, probe and artificial light. This was performed without informing children about oral examination and intervention dates. Dental Plaque was assessed using Plaque Index (PI) for Silness and Löe [7]. Gingival health was assessed using Gingival Index (GI) for Löe and Silness [7]. Blinding of the intervention was not possible, because it’s obvious to the investigator (S.B) who examined the children which cluster they were in. Realistically, it was going to be difficult to hide this information from children too.

**Fig. 3:**
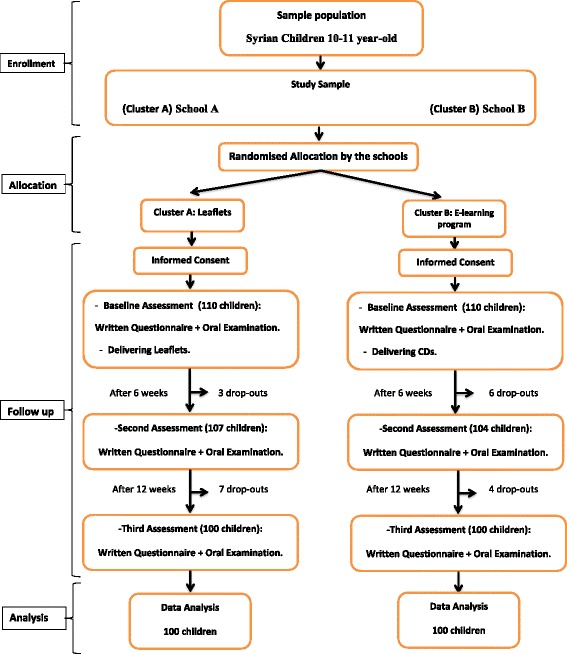
CONSORT diagram showing the flow of participants through each stage of the randomized trial

After collecting the baseline data, oral health educational tools were provided to subjects in which leaflets were given to children in Leaflet cluster, whilst children in E-learning cluster were provided with CDs which contained instructions on how to access the website via the link www.oralhealthforchildren.com. The level of oral health knowledge, plaque accumulation and gingival status were also re-evaluated after a period of six weeks and also after twelve weeks by the same examiner. The period of experiment was limited by the length of the school trimester in Syria which lasts 3 months (Fig. 3).

### Statistical analysis

The primary outcome was a change in oral health knowledge during the 12 weeks of the study in the two clusters. Secondary outcomes included changes in plaque accumulation and gingival health during the period of the study in the clusters. Data was entered in Microsoft Excel 2010, and statistically analyzed using the software SPSS 19.0. Descriptive statistical analysis was carried out. One way repeated measures ANOVA and post hoc Bonferroni test were used to compare the mean differences of study parameters (Oral health knowledge, PI scores and GI scores) within the same cluster. Between the two clusters, independent samples t-test was used to compare the mean differences of parameters evaluated at baseline, 6 weeks and 12 weeks. Level of significance and confidence interval were set at 5 and 95%, respectively.

## Results

About 220 schoolchildren aged 10–11 years were included in this study, and then there was a drop-out of 20 subjects. A total of 200 children (91 boys and 109 girls) were then included in the study in which, 100 children allocated to Leaflet cluster and the other 100 were grouped in E-learning cluster. The mean age for the study population was 10.74 ± 0.44 (Table 1).

**Table 1:** Age and gender distribution of children studied

	*Gender*			
Cluster	Male *N* (%)	Female *N* (%)	Total *N* (%)	Age Mean ± SD
Leaflet Cluster	43 (43)	57 (57)	100 (50)	10.69 ± 0.47
E-learning Cluster	48 (48)	52 (52)	100 (50)	10.80 ± 0.40
Total	91 (45.5)	109 (54.5)	200 (100)	10.74 ± 0.44

### Oral health knowledge scores

At the start of the study, the mean knowledge scores of children in the two clusters did not present any statistical significant differences (*P* = 0.73) (Table 2). After the intervention, the mean knowledge score was 82.87 ± 10.69 at 6 weeks and 89.12 ± 8.16 at 12 weeks in Leaflet cluster, while E-learning cluster showed values of 72.16 ± 10.25 at 6 weeks and 74.66 ± 8.98 at 12 weeks. Comparison of the baseline values with their respective post-intervention knowledge scores illustrated a statistically significant (*P* < 0.05) increase in knowledge for both clusters (Table 2). However, children in Leaflet cluster had significantly better knowledge than those in E-learning cluster at 6 weeks (*P* < 0.001) and at 12 weeks (*P* < 0.001) (Table 2). Further analysis using independent samples t-test revealed that the difference in knowledge gain was statistically significant between the two clusters, and that the increase was higher in Leaflet cluster than in E-learning cluster (34.19 ± 11.35 versus 19.60 ± 9.75, respectively, *P* < 0.001).

**Table 2:** The intracluster and intercluster comparison of oral health knowledge score between the two clusters

	Knowledge score Mean ± SD			
Cluster	Baseline	6 weeks	12 weeks	*P value*
Leaflet Cluster	54.94 ± 12.74	82.87 ± 10.69	89.12 ± 8.16	*F* = 665.67; *P* < 0.001*
E-learning Cluster	55.50 ± 9.93	72.16 ± 10.25	74.66 ± 8.98	*F* = 223.39; *P* < 0.001*
Significance	*t* = − 0.35; *P* = 0.73**	*t* = 7.24; *P* < 0.001**	*t* = 11.92; *P* < 0.001**	

*One way repeated measures ANOVA was applied to compare the mean differences of knowledge score within the same cluster

**Independent samples t-test was applied to compare the mean differences of knowledge score between the two clusters at baseline, 6 weeks and 12 weeks

### PI scores

PI scores in the two clusters were similar with no statistically significant difference at baseline (*P* = 0.17) (Table 3). After the oral health education, the mean PI score was 1.06 ± 0.33 at 6 weeks and 0.85 ± 0.35 at 12 weeks in Leaflet cluster. On the other hand, in E-learning cluster it was 1.31 ± 0.39 and 1.21 ± 0.40 at 6 weeks and 12 weeks, respectively.

**Table 3:** The intracluster and intercluster comparison of plaque index score between the two clusters

	PI score Mean ± SD			
Cluster	Baseline	6 weeks	12 weeks	*P* value
Leaflet Cluster	2.25 ± 0.43	1.06 ± 0.33	0.85 ± 0.35	F = 733.57; *P* < 0.001*
E-learning Cluster	2.33 ± 0.38	1.31 ± 0.39	1.21 ± 0.40	F = 427.62; *P* < 0.001*
Significance	*t* = − 1.39; *P* = 0.17**	*t* = − 4.81; *P* < 0.001**	*t* = − 6.82; *P* < 0.001**	

*Test applied: One way repeated measures ANOVA

**Test applied: Independent samples t-test

Within the cluster comparisons using one way repeated measures ANOVA, a significant improvement of oral health with decreased PI scores in both clusters was observed (Table 3). As for the intercluster comparison using independent samples t-test, Leaflet cluster had significantly lower PI scores than E-learning cluster at 6 weeks (*P* < 0.001) and at 12 weeks (*P* < 0.001) (Table 3).

### GI scores

At baseline, the differences in the mean GI scores between Leaflet cluster and E-learning cluster were not statistically significant (*P* = 0.12) (Table 4). After the intervention, the mean GI scores were 0.88 ± 0.25 at 6 weeks and 0.74 ± 0.22 at 12 weeks in children in Leaflet cluster. The mean GI scores in children related to E-learning cluster were 1.17 ± 0.25 at 6 weeks and 1 ± 0.25 at 12 weeks. Comparison between the baseline values (1.76 ± 0.36 in Leaflet cluster versus 1.83 ± 0.34 in E-learning cluster) and their respective post-intervention GI scores, revealed a statistically significant decrease in GI scores in both clusters (Table 4). Also, the independent t-test for intercluster comparison showed that Leaflet cluster had lower GI scores than E-learning cluster, and this difference was statistically significant at 6 weeks (*P* < 0.001) and at 12 weeks (*P* < 0.001) (Table 4).

**Table 4:** The intracluster and intercluster comparison of gingival index score between the two clusters

	*GI score Mean ± SD*			
Cluster	Baseline	6 weeks	12 weeks	*P value*
Leaflet Cluster	1.76 ± 0.36	0.88 ± 0.25	0.74 ± 0.22	*F* = 803.33; *P* < 0.001*
E-learning Cluster	1.83 ± 0.34	1.17 ± 0.25	1 ± 0.25	*F* = 441.12; *P* < 0.001
Significance	*t* = − 1.57; *P* = 0.12**	*t* = − 8.34; *P* < 0.001**	*t* = − 7.92; *P* < 0.001**	

*Test applied: One way repeated measures ANOVA

**Test applied: Independent samples t-test

Due to the educational nature of the intervention, no untoward effects were anticipated nor observed.

## Discussion

To our knowledge, this study is the first of its kind to investigate the role of E-learning instructions in improving oral health in children, and to compare it with the traditional educational leaflets in school children in Syria. The results of this school-based educational intervention were found to be effective for short term improvement of oral health knowledge, gingival health and in decreasing plaque levels in primary school children.

The target group for the specific oral health education was the primary school children because of their consumption of large amounts of sugars and soft drinks. Children aged 10–11 years were selected since they can, at this age, do logic thoughts, can realize the cause-result interaction, and explore everything. Younger children possibly would not be able to present those skills [8].

Since the semester in Syrian school lasts for three months, it was necessary to follow up children at 6 weeks and 12 weeks of oral health education. In addition, the amount of time and frequency with which children were exposed to the two different educational tools were similar to previous works [9–12].

Results of the present study suggest that baseline knowledge scores, the mean plaque index and mean gingival index scores in the two clusters were almost similar with no statistical differences, since the children included were in the same age group, similar socioeconomic status and did not receive any previous dental educational program. However, a statistically significant increase in knowledge score was seen in the two clusters after health education programs. This improvement can be attributed to the health messages delivered interactively to children as a short story, in simple language, with colorful images, quizzes as well as videos, so the children could get useful information in an easy and entertaining way. In addition, the study sample expressed a desire to discover the new educational materials provided to them, either through leaflets or E-program. The results of the present study were in accordance with other studies [10, 13–19] aiming at improving knowledge and health behavior. The findings of this study were also consistent with a study that claimed that using educational printed materials and websites had a significantly positive effect on the acquisition of knowledge [20]. In our study, after the educational program, better results in knowledge score were found in Leaflet cluster as compared to E-learning cluster at 6 weeks and 12 weeks. This finding can be attributed to the fact that introduction of web-based educational programs to disseminate health education among school children, is still in its embryonic stage in Syria, where parents and children still depend on textbooks, TV programs, lectures or leaflets as sources of health awareness. The idea of introducing internet-based health education to schools appeared to be a new and unfamiliar approach to children and their parents. Consequently, some children were not computer-literate to gain access to the website, and some children found difficulties in using smart phones to access the entire contents of the website. Moreover, children in E-learning cluster were hampered by the slow connection speeds when using sound, graphics and video files, and other technical problems were also reported. Due to these challenges, this study can be considered as a baseline study in which future work could be undertaken after a period of time to evaluate the development of knowledge in the field of technology and its implementation in improving oral health care. On the other hand, leaflets were able to reach a large segment of school children regardless of the frequency of dental visits, socioeconomic status, possession of computers and internet network at their homes, and other technical problems related to using website as an educational method to disseminate information among school children in Damascus city. These findings were in accordance with previous study [21] which found that leaflets are a cost-effective way to spread awareness about prevention of dental caries. This is particularly true in developing countries where budgetary allocations are restricted and resources to disseminate electronic educational materials are limited and often hindered with technical difficulties. In agreement with our results, previous studies reported that leaflets are good educational method that can raise awareness and deliver health messages to members of the community [22, 23]. In contrast, our findings were different from another study conducted in Germany which found that web-based multimedia program was more effective than traditional print-based self-study by medical students [24].

As for the oral hygiene status, the mean PI and GI scores were significantly lower in both clusters, and this could be attributed to the fact that the use of animated colorful pictures, videos and quizzes in the E-learning program, similarly, the style of short story in simple language and pictorial sketches in the leaflet can help children to understand the concepts of oral health better. Besides emphasizing some immediate gains from good oral hygiene such as fresh breath, clean and white teeth and attractive appearance, were key aspects for motivating these children and creating an interest to modify their behavior. Results of the present study were comparable with many studies [9, 11, 13, 25–27] depicting the impact of school dental health education programs which resulted in significant improvement in oral hygiene of school children after imparting dental health education. Another study found no significant reduction in plaque scores of the children after short-term dental health education program [28].

In comparing the two clusters, highest improvement in oral hygiene status was seen in Leaflet cluster. A reason for notable lack of improvement of oral hygiene in E-learning cluster may be that children in this cluster were hampered by many difficulties that mentioned earlier, and consequently they had not achieved a notable behavior change related oral hygiene. Consistent with our findings, other previous studies found that children in the leaflets group showed positive results reflected on their daily oral health practices compared with other study groups [12, 29]. In accordance with interventions in Iran [11] and Brazil [21], the present results bring to light the importance of educational leaflets in improving oral health status and behavior of school children. This is especially true in countries with a developing oral health care system, where the need is to find a suitable educational program without relying upon costly professional input.

This study provides valuable insight regarding the effectiveness of dental health education among Damascus City’s school-aged children. However, there are some limitations. The length of the study was limited to a time frame of 3 months, which may be considered a relatively short period, so the permanence of the impact requires more longitudinal research. Additionally, the present study has been conducted in a small geographic area, and its results will be better validated via multicenter studies. However, the study was conducted at public schools which represent the largest segment of schools in Damascus city, and that will make it more generalizable.

## Conclusions

From the results observed, it can be concluded that short term oral health education programs may be useful in improving oral hygiene practices in children. Educational instructional leaflets are appropriate effective economic tools for improving oral and gingival health among Syrian children when compared to E-learning program, and they can be suggested as educational tools in school-based oral health education programs with more fruitful outcomes. How long the benefit will be retained is an important question in all health education programs. Further longitudinal studies to study the retention of knowledge and oral hygiene practices are therefore required and crucial.

## Additional file

Additional file 1: Oral Health Questionnaire. (DOCX 1121 kb)

## Abbreviations

CD: Compact disc
CONSORT: Consolidated Standards of Reporting Trials
DMFT: Decayed missing filled permanent teeth
dmft: Decayed, missing, filled primary teeth
GI: Gingival index
PI: Plaque index
SD: Standard deviation
