# Supplementary material for: E-learning or educational leaflet: does it make a difference in oral health promotion? A clustered randomized trial
Source: BMC Oral Health. 2018 May 10;18:81. doi: 10.1186/s12903-018-0540-4 (PMC5946495; doi:10.1186/s12903-018-0540-4)
Supplement: Supplementary file 1 — Oral Health Questionnaire. (DOCX 1121 kb) [file 12903_2018_540_MOESM1_ESM.docx]

**Oral Health Questionnaire**

**Name: Age: Gender:**

**School Name:**


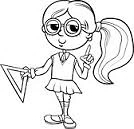


**Hello my friends… Can you help me answering the following questions?**

**Select one answer to each of the following questions:**

1. How many are the baby teeth?

- 10 teeth
- 15 teeth
- 20 teeth
- 32 teeth

1. How many are the adult teeth?

- 10 teeth
- 15 teeth
- 20 teeth
- 32 teeth

1. What is tooth decay?

- Little hole caused by germs
- Staining of the teeth
- Inflammation of the gum
- I do not know

1. How often do you brush your teeth?

- I do not brush my teeth
- Once a day
- Twice a day
- After every meal

1. What do you use for cleaning your teeth?

- Brush
- Brush + Toothpaste + Dental floss
- Mouthwash
- Toothpicks

1. How long does it take to brush your teeth?

- Less than one minute
- One minute
- Two minutes
- More than two minutes

1. When should you get a new toothbrush?

- Every year
- Every 3-4 year
- I do not change my brush
- I do not know

1. How often do you eat sweets and soft drinks?

- I do not eat sweets and soft drinks
- Less than three times a day
- Three times a day
- More than three times a day

1. If you eat sweets, when do you usually have them?

- With the main meals ( Breakfast- Lunch- Dinner)
- Between the main meals
- Before going to bed
- There is no specific time

1. What does eating too many sweets lead to?

- Sweets never affect the teeth
- Sweets prevent tooth decay
- Eating too much sweets can lead to tooth decay
- I do not know

1. How often do you visit your dentist?

- Regularly twice a year
- Occasionally
- When I have dental pain
- I never visit a dentist

1. What does plaque mean?

- Hard debris on the teeth
- Staining of the teeth
- Soft debris on the teeth
- I do not know

1. What does dental plaque lead to?

- Your teeth become more shiny
- Tooth decay and inflammation of the gum
- Staining of the teeth
- I do not know

1. What is fluoride?

- Fluoride strengthens the teeth
- Fluoride harms the teeth
- Fluoride prevents tooth decay
- I do not know


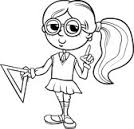


**Now, my friends, let’s put the following drinks in the proper container ☺**

Milk – Sweetened drinks – Lemon juice – Water – Orange juice – Soft drinks


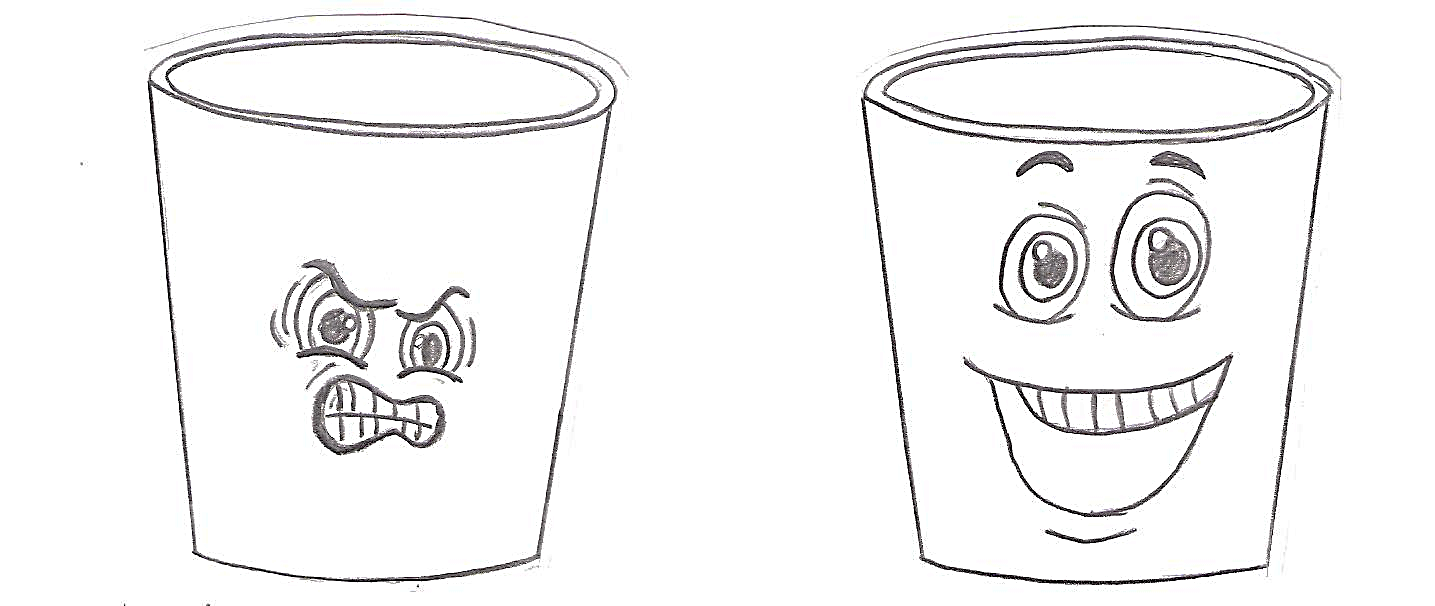


Harmful drinks for my teeth Good drinks for my teeth


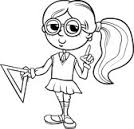


**My friends, let’s put the following foods in the proper container ☺**

Carrot – Banana – Biscuit – Chips – Cheese – Cookies – Chocolate – Apple – Ice cream – Candy – Cucumber

**
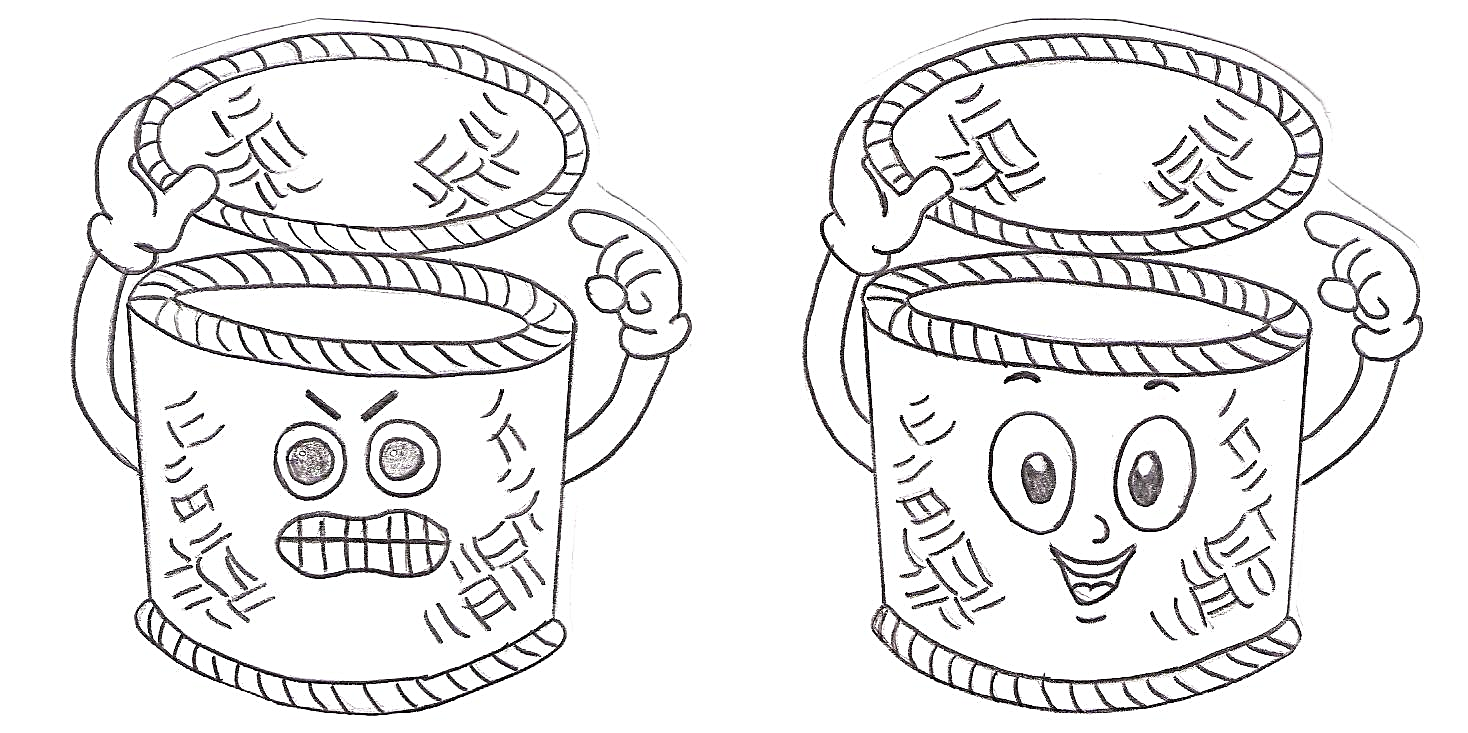
**

Harmful foods for my teeth Good foods for my teeth
